# Empathy Modulates the Activity of the Sensorimotor Mirror Neuron System during Pain Observation

**DOI:** 10.3390/bs13110947

**Published:** 2023-11-17

**Authors:** Julio Plata-Bello, Nicole Privato, Cristián Modroño, Yaiza Pérez-Martín, África Borges, José Luis González-Mora

**Affiliations:** 1 Department of Neurosurgery, Hospital Universitario de Canarias, S/C de Tenerife, 38320 La Laguna, Spain; 2 Cognitive Neuroscience Research Group, University of La Laguna, 38320 La Laguna, Spain; 3 Department of Neurology, Hospital Universitario de Canarias, S/C de Tenerife, 38320 La Laguna, Spain; 4 Department of Physiology, Faculty of Medicine, University of La Laguna, 38320 La Laguna, Spain; 5 Department of Clinical Psychology, Psychobiology and Methodology, University of La Laguna, 38320 La Laguna, Spain

**Keywords:** mirror neuron system, pain observation, empathy

## Abstract

Aim: The aim of this study is to analyze the brain activity patterns during the observation of painful expressions and to establish the relationship between this activity and the scores obtained on the Interpersonal Reactivity Index (IRI). Methods: The study included twenty healthy, right-handed subjects (10 women). We conducted a task-based and resting-state functional magnetic resonance imaging (fMRI) study. The task involved observing pictures displaying painful expressions. We performed a region of interest (ROI) analysis focusing on the core regions of the sensorimotor mirror neuron system (MNS). Resting-state fMRI was utilized to assess the functional connectivity of the sensorimotor MNS regions with the rest of the cortex using a seed-to-voxel approach. Additionally, we conducted a regression analysis to examine the relationship between brain activity and scores from the IRI subtests. Results: Observing painful expressions led to increased activity in specific regions of the frontal, temporal, and parietal lobes. The largest cluster of activation was observed in the left inferior parietal lobule (IPL). However, the ROI analysis did not reveal any significant activity in the remaining core regions of the sensorimotor MNS. The regression analysis demonstrated a positive correlation between brain activity during the observation of pain and the “empathic concern” subtest scores of the IRI in both the cingulate gyri and bilateral IPL. Finally, we identified a positive relationship between the “empathic concern” subtest of the IRI and the functional connectivity (FC) of bilateral IPLs with the bilateral prefrontal cortex and the right IFG. Conclusion: Observing expressions of pain triggers activation in the sensorimotor MNS, and this activation is influenced by the individual’s level of empathy.

## 1. Introduction

Empathy refers to the ability to perceive and understand the emotions and thoughts of others [1], and involves an individual’s reactions to the experiences of others [2]. Empathy can also be defined as the capacity to emotionally connect with others, irrespective of sharing the same situation [3]. One universally unpleasant human experience is pain, which transcends various cultures. Pain is defined as an unpleasant sensory and emotional state associated with, or resembling, actual or potential tissue damage (source: International Association for the Study of Pain—https://www.iasp-pain.org/). Empathy for pain involves the processes of perceiving, assessing, and responding to the pain experienced by others [4,5]. The observation of pain in others often generates a negative emotional or cognitive state, which is generally considered aversive [6].

Brain processes involved in empathy for pain have been extensively studied. It is assumed that the processes of pain and empathy for pain share common brain networks, predominantly encompassing areas associated with emotions [1,7]. Some studies have shown that pain primarily activates the anterior cingulate cortex and the insula [8,9,10,11]. However, activation of motor and somatosensory brain regions has also been noted during pain processing [7,11]. Conversely, observing pain or pain expressions activates a multitude of brain regions in healthy individuals. These regions are distributed across the frontal cortex (including the inferior frontal gyrus (IFG), cingulate gyrus, and premotor areas), temporal cortex, occipital cortex, insula, and several subcortical nuclei (such as the thalamus, putamen, caudate, and amygdala) [6,11].

One functional network potentially associated with the processing of observed pain is the mirror neuron system (MNS). The MNS is a network in the brain believed to contain mirror neurons, which activate not only when an action is performed but also when it is observed [12,13,14]. While the primary functions attributed to the MNS in social cognition are action understanding and imitation [15], a significant body of literature supports its involvement in empathy. The MNS has been dichotomized into the sensorimotor and emotional MNS [16]. The sensorimotor MNS is primarily engaged in motor functions and consists of core regions in the bilateral IFG and inferior parietal lobule (IPL). The emotional MNS, on the other hand, plays a role in the expression, experience, and perception of emotional displays on faces and bodies [17]. Affective empathy is predicated on the ability of social stimuli to trigger visceromotor actions in the observer, in addition to somatomotor actions [18,19]; this network includes regions such as the anterior cingulate cortex (ACC), amygdala [20], and insula [21,22]. Nevertheless, somatomotor processing also occurs during emotional processing, suggesting that the sensorimotor MNS may also activate during empathic processes. Furthermore, subcortical connectivity supports interaction between the two MNS components. The ACC and the amygdala are structurally connected with the premotor cortex (including the IFG) [23], while the insula has extensive connections with the premotor cortex and the IPL [24].

The activation of brain regions within the sensorimotor MNS during pain observation remains a subject of investigation. Apart from the activation of cortical areas also engaged by the first-hand experience of pain (i.e., the ACC and the insula) [25], some authors have reported the activation of core regions of the sensorimotor MNS during pain observation [26,27]. This activation has been associated with the embodiment of the observed action, although it has also been suggested that it may be linked to the relationship between the MNS and empathy [28]. Even in the absence of actual pain stimuli, certain studies indicate that the sensorimotor cortex is activated [29,30]. However, these studies did not measure empathic ability [1,31], and as a result, the connection between sensorimotor MNS activity and empathic ability remains undefined.

The primary objective of this study is to delineate the brain activity patterns during the observation of painful expressions, with a particular focus on the analysis of sensorimotor MNS activity. Additionally, we aim to establish a relationship between this activity and Interpersonal Reactivity Index (IRI) scores as a measure of empathic ability. We will also investigate potential associations between IRI sub-scores and the functional connectivity of core sensorimotor MNS regions. We hypothesize that the observation of painful expressions may activate core regions of the sensorimotor MNS. In terms of the hypothetical relationship between the MNS and empathy, we expect that the activity in sensorimotor MNS regions among participants will correlate with their level of empathy, as evidenced by IRI scores, and that this relationship may also be observed in the functional connectivity of MNS regions.

Understanding how the brain processes the pain of others and its relationship with empathy is of paramount importance. This knowledge can shed light on the behaviors of healthcare workers and caregivers and may facilitate the planning of interventions to enhance empathic scores through modulation of extrinsic neural networks.

## 2. Methods

### 2.1. Subjects

Twenty healthy, right-handed (Edinburgh Handedness Inventory [32] < 25) subjects were selected (10 women), with an average age of 23.15 (SD = 2.45). Participants did not present any previous history of neurological or psychiatric disease. Written informed consent was explained and signed. The study was approved by the University of La Laguna Ethics Committee according to the Declaration of Helsinki.

### 2.2. Data Acquisition and Processing

The experimental data were collected at the Magnetic Resonance for Biomedical Research Service, University of La Laguna. Two functional runs were conducted: one for task-based fMRI and another for resting-state fMRI.

For task-based functional imaging, we employed a 3 T General Electric scanner (Milwaukee, WI, USA) with an echo planar imaging gradient echo sequence and an 8-channel head coil. The acquisition parameters were as follows: TR = 3000 ms, TE = 21 ms, flip angle = 90°, matrix size = 64 × 64 pixels, 57 slices/volume, interslice gap = 1 mm, and slice thickness = 3 mm. The slices were aligned to the anterior commissure–posterior commissure line, encompassing the entire cranium. To ensure tissue steady-state magnetization, we performed 18 s of dummy scans before the functional scanning.

For the resting-state fMRI, we used the same equipment with the following parameters: TR = 2000 ms, TE = 22.1 ms, flip angle = 90°, matrix size = 64 × 64 pixels, 36 slices/volume, interslice gap = 1 mm, and slice thickness = 4 mm, maintaining the same slice alignment.

To provide an anatomical reference, we obtained a whole-brain three-dimensional structural image using a 3D fast spoiled gradient–recalled pulse sequence with the following acquisition parameters: TR = 10.4 ms, TE = 4.2 ms, flip angle = 20°, matrix size = 512 × 512 pixels, 0.5 × 0.5 mm in-plane resolution, and slice thickness = 2 mm.

After thorough image artifact inspection, the task-fMRI data underwent preprocessing and analysis using Statistical Parametric Mapping software SPM12 from the Wellcome Trust Centre for Neuroimaging (http://www.fil.ion.ucl.ac.uk/spm/). The images were spatially realigned, unwarped, and normalized to the Montreal Neurological Institute (MNI) space following standard SPM12 procedures. The normalized images had a resolution of 2 × 2 × 2 mm and were smoothed with a full width at half maximum (FWHM) 8 × 8 × 8 Gaussian kernel.

Conversely, the resting-fMRI data underwent similar preprocessing using SPM12, except for the smoothing step, which utilized an FWHM 6 × 6 × 6 Gaussian kernel. Additionally, we discarded the first 10 images to eliminate signal equilibration effects. Subsequently, we removed sources of spurious variance through linear regression, incorporating signals from the ventricular system, white matter, and the entire brain, along with the six parameters obtained from rigid body head motion correction. Finally, the signal was linearly detrended, and a temporal band-pass filter was applied (0.01 Hz < f < 0.08 Hz). All participants performed a questionnaire to measure the Interpersonal Reactivity Index (IRI), consisting of 28 items answered on a 5-point Likert scale ranging from “Does not describe me well” to “Describes me very well” [2]. The measure had three subscales, which were: “Perspective Taking” (PT), “Fantasy” (FS), “Empathic Concern” (EC), and “Personal Distress” (PD). According to Davis MH (1983), PT refers to the tendency to spontaneously adopt the psychological point of view of others; FS shows respondents’ tendencies to transpose themselves imaginatively into the feelings and actions of fictitious characters in books, movies, and plays; EC assesses “other-oriented” feelings of sympathy and concern for unfortunate others; and PD measures “self-oriented” feelings of personal anxiety and unease in tense interpersonal settings [2].

### 2.3. Study Design

**Figure 1:**
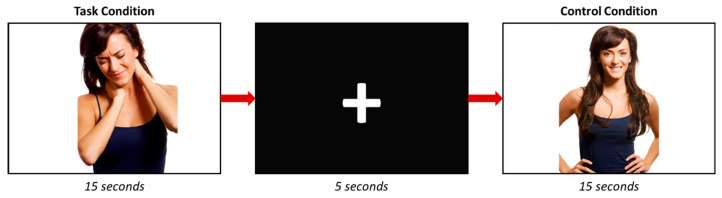
Scheme of the task-fMRI experiment.

During the task-fMRI run, participants were asked to visualize a series of photographs where an actor or an actress appears with a facial expression of pain (Figure 1). Each photograph appears for 5 s in a block of three pictures; thus, each block lasts 15 s. A total of 6 different pictures with painful facial expressions were included in the study. The control condition consisted of photographs where the same actors appeared without showing any pain expression. In this regard, 6 photographs were also included, and each appears during 5 s in a block of 15 s (3 photographs/block). Six task and 6 control blocks were performed, and they were separated by a five-seconds black screen with a white cross in its center. To sum up, in one block, 3 images (with or without pain) were displayed. The images appeared randomly within each corresponding block (with or without pain). Subsequently, the images were repeated across blocks.

The photographs were chosen following a brief validation process, which involved two researchers (J.P.B. and N.P.) selecting a set of 50 image pairs, each depicting a subject with and without a pain gesture. Subsequently, these image pairs were presented to a group of 50 participants (with a mean age of 22.1, SD = 2.25), who evaluated the images using a Likert scale that ranged from 1 (indicating no pain) to 5 (representing severe pain). We analyzed scores of images depicting pain versus those not depicting pain using a non-parametric test (Wilcoxon’s W). Significance was assigned when the corrected *p*-value was below 0.05 (False Discovery Rate (FDR) < 0.05). Among the 50 image pairs, 39 demonstrated significantly higher scores for images depicting pain (FDR < 0.05). We chose 6 image pairs with the lowest standard deviation (SD < 1.7), indicating less inter-observer variability.

During the resting-fMRI run, participants were instructed to keep their eyes closed and not to think about anything throughout the run. A questionnaire after the scan confirmed that none of the subjects fell asleep.

### 2.4. Simple T Contrasts

A block design in the context of a general linear model was used for the individual subject analyses (first level) to look for differences in brain activity during the periods of pain expression observation and the control condition; the contrast in the analysis was pain observation > control. Only voxels in grey matter locations were considered. The first-level contrast images were then used in a random-effects group analysis (second level). The age, gender, and Edinburgh Handedness Inventory Score [32] of participants were included as covariates. Group analysis was performed using the random effect approach, using a one-sample *t*-test (Family Wise Error (FWE) = 0.05) with a minimum cluster of twenty voxels.

Furthermore, a regression analysis between the brain activity during the facial pain expression observation and each IRI’s subtest score was performed. The scores of the four subscales were included as covariates, and the association between the brain activity during pain observation and each subscale score was determined (*p*-uncorrected < 0.001).

### 2.5. Region of Interest Analysis

Regarding the aim of this work to analyze the activity in the sensorimotor MNS regions during facial pain expression observation, a region of interest (ROI) analysis in the core regions of this network was performed. Masks for each core region (i.e., IFG and IPL) of both brain hemispheres were generated using WFU Pickatlas (https://www.nitrc.org/projects/wfu_pickatlas/) [33]. The supramarginal gyrus (SMG) and the angular gyrus (AG) were included as ROIs because they have also shown mirror properties and they are normally considered as a part of the IPL mask, as other authors have previously assumed [34,35]. ROIs data were extracted using the MarsBaR 0.44 toolbox (http://marsbar.sourceforge.net/). A simple T contrast was performed to identify the activity in the sensorimotor MNS regions during the observation of facial pain expressions. Furthermore, regression analyses between the activity in each of the specified ROIs and the IRI’s subscales scores were performed. The four IRI’s subscales scores were included in the same model. Statistical significance for any contrast of the ROI analysis was considered when the corrected *p*-value was below 0.05.

### 2.6. Resting-State Analysis: Functional Connectivity

Functional connectivity (FC) between proximal or distant brain regions can be inferred from inter-regional cross-correlations of the BOLD signal at rest [36]. Using the Resting-State fMRI Data Analysis Toolkit (REST) version 1.8 [37], an FC analysis was performed using the seed-to-voxel approach. ROIs (seeds) were the same as described in the previous section, but to simplify the presentation of the results, the SMG and the AG masks were integrated with the IPL as a single ROI. Thus, 4 ROIs were analyzed for FC: the left IFG and extended left IPL; the right IFG and extended right IPL. Individual z-score maps were obtained, and after that, a one-sample *t*-test was performed on them. The statistical significance threshold was set to *p* < 0.01 with a cluster size of 40 voxels, using the REST AlphaSim [37], which corresponded to a corrected *p* < 0.05. Moreover, a regression analysis was performed between the FC of each ROI and each of the IRI’s subscales scores. Statistical significance for any contrast of the ROI analysis was considered when the corrected *p*-value was below 0.05.

## 3. Results

### 3.1. Brain Activity during the Observation of Pain Expressions

**Figure 2:**
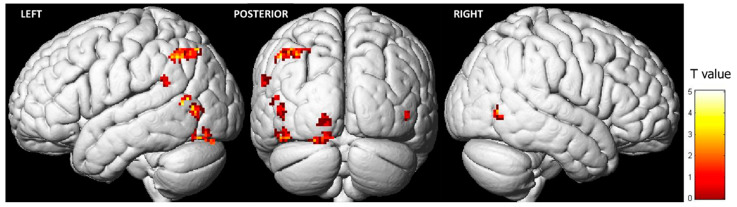
Brain activation during the observation of pain expression (contrast: pain expression vs. control) (FWE = 0.05; k = 20).

Observing pain expressions led to heightened activity in specific regions of the frontal, temporal, and parietal lobes (Table 1; Figure 2). The most significant cluster of activation (FWE < 0.05) was in the left inferior parietal lobule (IPL). Additionally, key clusters of activation in the occipital lobe were found in the left middle occipital gyrus (MOG) and the left lingual gyrus (LG). Lastly, the primary cluster of activity in the temporal lobe was situated in the left superior temporal gyrus (STG). In the right hemisphere, higher activity was observed exclusively in the right middle temporal gyrus (MTG) during the observation of pain expressions. Conversely, the opposite contrast (i.e., control condition > pain expression faces) did not reveal any differences.

As this study primarily focuses on investigating the sensorimotor MNS activity during the observation of pain expressions, we conducted a region of interest (ROI) analysis in the core MNS regions considered (i.e., IPL, SMG, AG, and IFG, in both hemispheres). The left IPL (t = 3.35, corr-*p* = 0.014), the left SMG (t = 3.63, corr-*p* = 0.008), and the left AG (t = 3.94, corr-*p* = 0.004) exhibited significantly higher activation during the observation of pain facial expressions. None of these regions in the right hemisphere displayed statistically significant differences, even when uncorrected *p*-values were considered (right IPL: t = 1.21, corr-*p* = 0.645; right SMG: t = 0.64; corr-*p* = 0.914; right AG: t = 0.96, corr-*p* = 0.787) (Supplementary Table S1). Concerning the frontal component of the MNS network (i.e., the inferior frontal gyrus (IFG)), none of these regions in both hemispheres showed differences during the observation of pain facial expressions (left IFG: t = 2.31, corr-*p* = 0.127; right IFG: t = 1.05, corr-*p* = 0.738). However, in the case of the left IFG, the uncorrected *p*-value of the ROI analysis demonstrated statistical significance (unc-*p* = 0.016), which did not maintain significance when corrected *p*-values were considered (Supplementary Table S1).

It is worth noting that gender-specific differences were not identified in either the group or the ROI analysis (Supplementary Table S1).

### 3.2. Regression Analysis between Brain Activity during Pain Expression Observation and IRI Subscale Scores

**Figure 3:**
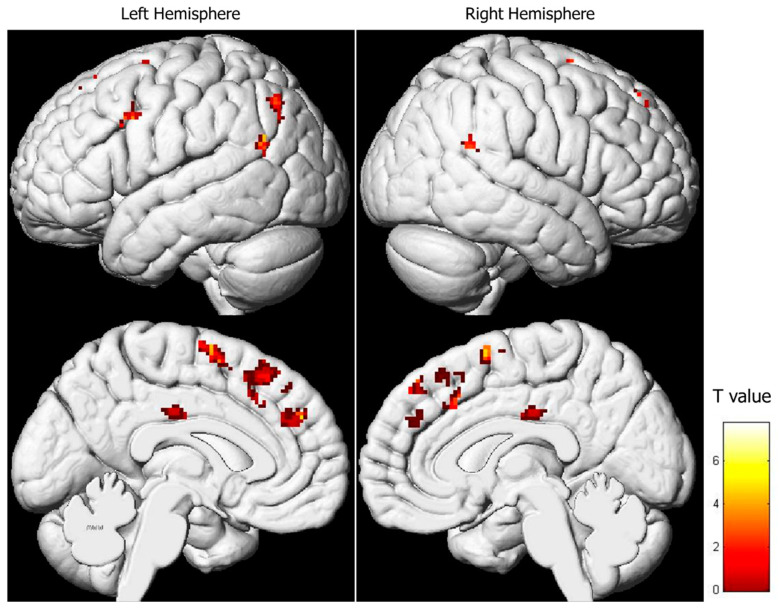
Regression analysis between the activity during pain facial expression observation and the results of the “Empathic Concern” subscale (uncorrected-*p* = 0.001; k = 20).

**Figure 4:**
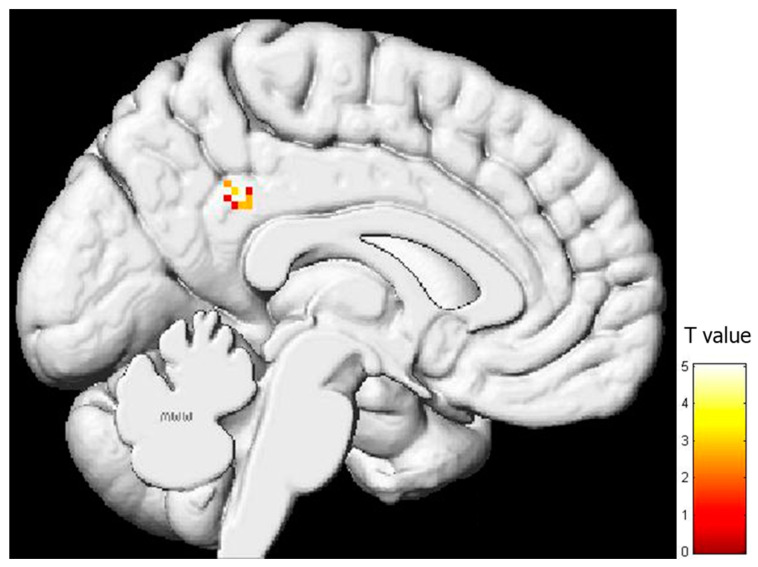
Regression analysis between the activity during pain facial expression observation and the results of the “Personal Distress” subscale (uncorrected-*p* = 0.001; k = 20).

As explained in the methods section, the IRI comprises four subscales: “Perspective Taking” (PT), “Fantasy” (FS), “Empathic Concern” (EC), and “Personal Distress” (PD). No significant relationship was found between brain activity during pain expression observation and the PT and FS subscale results. However, a positive correlation emerged between brain activity during pain expression observation and EC scores, which was manifested in the superior and medial frontal lobes, as well as the parieto-temporal regions (Table 2, Figure 3). In essence, higher activity in both the right and left superior and medial frontal gyri (comprising the supplementary motor area) and in both cingulate gyri corresponded to higher EC subtest scores. Similarly, the parieto-temporal regions (including both IPLs) exhibited such a positive relationship. Conversely, a negative correlation was observed between activity in the left posterior cingulate gyrus and scores on the PD subtest (Table 3, Figure 4).

**Figure 5:**
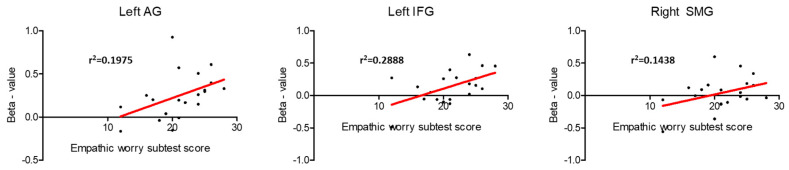
Relationship between brain activity during pain facial expression observation and the score in the “Empathic Concern” IRI’s subscale in the mirror neuron system core regions that showed statistical significance in the ROI analysis.

ROI analysis in the core regions of the sensorimotor MNS was also conducted in the regression analysis (Supplementary Tables S1 and S2). A positive association between brain activity during pain expression observation and EC subscale results was detected in the left AG (t = 3.35, corr-*p* = 0.033), the left IFG (t = 3.28, corr-*p* = 0.037), and the right SMG (t = 3.40, corr-*p* = 0.030) (Figure 5). No other connections were found between brain activity during the observation of pain facial expressions and the remaining IRI subscales in the MNS regions (Supplementary Tables S1 and S2).

### 3.3. Functional Connectivity of the Sensorimotor MNS Core Regions

**Figure 6:**
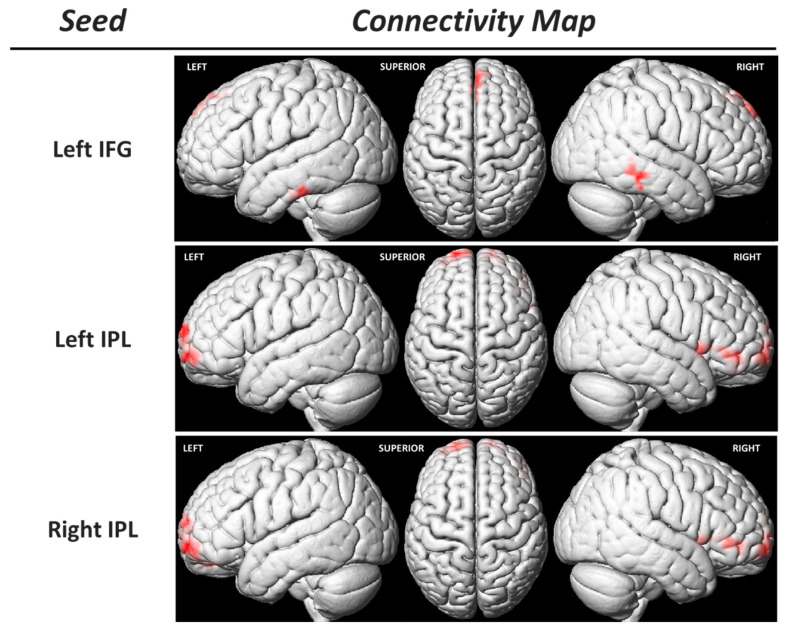
Functional connectivity (FC) analysis using the seed-to-voxel approach. The region of interest that constitutes the seed is indicated in the left column, and each FC map is in the right one (corrected *p*-value = 0.05). IFG: inferior frontal gyrus; IPL: inferior parietal lobule.

Considering the previous findings, we conducted a regression analysis between the functional connectivity (FC) of MNS core regions (using seed-to-voxel analysis) and the EC IRI subtest scores. This analysis revealed a positive correlation between the EC IRI subtest scores and the FC of bilateral IPLs with the bilateral superior frontal gyrus (prefrontal cortex) and the right IFG (Table 4, Figure 6). Additionally, a positive relationship was observed between the FC of the left IFG and the right MTG, left ITG, and the right supplementary motor area (SMA) with this subtest score (Table 4, Figure 6).

## 4. Discussion

This research comprised an fMRI experiment in which participants observed painful expressions. We identified activity in the left IPL, a core region of the sensorimotor MNS. Intriguingly, the extent of activity in this region exhibited a significant correlation with each participant’s level of empathy, a topic we will delve into in the subsequent discussion.

The presence of activity in the IPL would reflect the activity that occurs in the sensorimotor MNS during the observation of pain expressions. As mentioned in the introduction, some authors had already described the presence of activity in regions of the sensorimotor MNS during the observation of pain scenes [26,27], but the explanation given for this activity was the embodiment of gestures or movements/postures associated with pain. In other words, the activity of the sensorimotor MNS during the observation of pain has been solely attributed to the encoding of motor acts observed in association with the pain being observed. However, in the present study, we have observed that MNS activity is associated with the level of empathy exhibited by each subject. In other words, the activity of the left IPL during the observation of scenes depicting people in pain depends on the empathic concern displayed by each individual. This highlights how the activity of the sensorimotor MNS is modulated by individual characteristics unrelated to motor skills but related to the emotional or interpersonal intelligence of each individual [38].

However, the activity of the MNS has not been consistent within the whole brain network; rather, greater activity of the parietal component has been observed in comparison to activity in the frontal areas comprising the system. Some studies have already highlighted the existence of such differences. For instance, in the work of Montgomery KJ and Haxby J (2008), they concluded that social gestures performed with the hand lead to greater activation of the IPL, while facial expressions lead to greater activity in the IFG, indicating a differential representation of non-verbal communication types in the MNS [39]. Furthermore, parietal activity during the observation of movements greatly depends on the type of movement observed, as well as the type of object involved in the movement [40]. On the other hand, the recruitment of frontal areas during action observation is also related to the process of imitation, meaning there is greater activation of frontal regions when attempting to imitate what is observed after observation [41]. Given that the anticipation of pain is associated with activation, among other regions, of the IPL [42] and that this region is also involved in evaluating pain intensity [43], we believe that, in the context of perceiving pain in other individuals, the sensorimotor MNS plays a role and, specifically, translates into greater activity in parietal areas compared to frontal activity. However, this does not imply that no activity occurs in frontal areas or that this activity is modulated in the same way as parietal activity. In fact, we observed that, just as in parietal areas, there is a positive relationship between empathic concern and IFG activity during the observation of pain expressions. Therefore, the sensorimotor MNS is activated during the observation of pain expressions, with greater activity in parietal areas compared to frontal areas.

Additionally, it is worth noting that in this study, MNS activity is predominantly located in the left hemisphere. As indicated in the methodology section, all participants were right-handed, and there are reports of a certain left hemispheric dominance in MNS activity [44]. Furthermore, it has been observed that the mental simulation of sensory characteristics of pain experienced by others has a left hemispheric predominance [45]. On the other hand, the sensation of pain leads to greater activity in regions processing pain in the left hemisphere of the brain, while the right hemisphere is involved in the emotional component of pain [46]. Therefore, the greater activity of the left sensorimotor MNS and its positive correlation with empathic concern suggests that when an individual observes a pain scene, the regions that would be activated if they were experiencing the pain themselves (preferably in the left hemisphere) are activated to a greater extent, and this activation is even more pronounced in individuals who are more empathic, indicating that they “suffer” more from the observed pain.

Another intriguing finding of this study is the presence of a positive relationship between the degree of empathy and the functional connectivity (FC) of regions comprising the sensorimotor MNS both within themselves and with other brain regions. However, the most notable observation, perhaps, is the heightened FC between both IPLs and the prefrontal cortex (PFC) (Table 4, Figure 6). As suggested earlier, a greater degree of empathy may be linked to an enhanced ability to process and comprehend social and emotional signals from others. This heightened skill might manifest as increased connectivity among brain regions involved in perceiving and interpreting social signals, such as the IPL [47] and the PFC [48]. Furthermore, a positive relationship between empathy and functional connectivity within the supplementary motor area (SMA) was observed. The SMA is not only associated with the planning and execution of movements but may also be involved in emotional resonance, i.e., the capacity to experience emotions akin to those observed in others [49,50]. Greater functional connectivity with the left IFG could suggest that these regions collaborate to elicit a stronger empathetic response when observing the emotions of others. Given that the SMA is an area where the presence of mirror neurons has been demonstrated [51], future studies should investigate the role of this region in modulating the sensorimotor MNS, not only in relation to the comprehension of motor actions but also in connection with the emotional component that may be associated with them.

### Limitations

This study has several limitations that should be emphasized. First, despite having specifically studied the regions comprising the sensorimotor MNS, the fMRI tasks performed in the study only included observation, without a corresponding execution task (which in this case would involve producing a painful stimulus like the one observed) to confirm the existence of this mirror activity through a conjunction analysis. Additionally, besides the potential ethical limitations of inducing pain in a research context, evaluating MNS activity solely with observation tasks is a widely used approach in the MNS literature [52,53]. In this regard, to overcome this limitation, it would be interesting to analyze what happens in patients experiencing chronic pain, which could be the subject of future research. Secondly, it is important to consider the limitations of empathy assessment [54]. The use of the IRI as a two-factor model to determine cognitive and affective empathy has been widely criticized [55], with recommendations to analyze the four subscales separately; this is precisely what we have carried out in the present study. Therefore, despite the limitations of the measurement instrument, we have used it in a way that provides greater precision.

Finally, while our study focused on right-handed participants to reduce heterogeneity, it is worth noting that future investigations involving left-handed individuals could provide valuable insights into the neural correlates of empathy, particularly within the context of the MNS activity.

## 5. Conclusions

In conclusion, our study sheds light on the role of the sensorimotor MNS in processing observed pain expressions. We found that MNS activity, particularly in the left hemisphere, is modulated by individual differences in empathic concern. These findings expand our understanding of how empathy influences neural responses to pain in others.

## Figures and Tables

**Table 1:** Activation pattern during the observation of pain facial expressions (FWE = 0.05; k = 20). Only the contrast “pain facial expressions” > control is represented because the opposite contrast did not reach statistical significance.

Region	BA	x	y	z	T	Z	Voxels/Cluster
***OCCIPITAL LOBE***							
Left Middle Occipital Gyrus	19	−40	−68	4	4.69	3.71	54
Left Middle Occipital Gyrus	19	−48	−72	4	4.02	3.33	
Left Lingual Gyrus	18	−16	−82	−18	5.25	3.99	58
Left Inferior Occipital Gyrus	17	−10	−94	−14	5.17	3.96	
Left Middle Occipital Gyrus	18	−46	−78	−16	5.17	3.95	50
Left Lingual Gyrus	17	−10	−96	−8	4.70	3.71	28
Left Lingual Gyrus	17	−18	−94	−4	4.41	3.55	
***TEMPORAL LOBE***							
Left Superior Temporal Gyrus	22	−38	−58	12	5.50	4.12	59
Left Middle Temporal Gyrus	19	−52	−64	12	4.07	3.35	
Right Middle Temporal Gyrus	37	48	−66	0	4.07	3.36	28
***PARIETAL LOBE***							
Left Supramarginal Gyrus	40	−62	−46	28	4.03	3.33	23
Left Inferior Parietal Lobule	40	−42	−60	44	5.11	3.92	138
Left Inferior Parietal Lobule	40	−34	−68	48	4.23	3.45	

**Table 2:** Regression analysis between brain activity during pain facial expressions observation and “Empathic Concern” subscale (uncorrected-*p* = 0.001; k = 20). Only positive relationships are represented because the negative ones did not reach statistical significance.

Region	BA	x	y	z	T	Z	Voxels/Cluster	r^2^
***FRONTAL LOBE***								
Right Superior Frontal Gyrus	6, 8	8	6	66	6.16	3.76	39	0.228
		8	42	50	4.92	3.35	25	0.206
		2	40	44	4.59	3.21		0.198
Left Superior and Medial Frontal Gyrus	6, 8, 9	−6	28	60	5.57	3.58	87	0.219
		−6	30	52	5.21	3.45		0.212
		−2	20	52	4.62	3.23		0.199
		−4	−2	68	5.14	3.43	70	0.211
		−12	6	62	4.74	3.27		0.202
		−2	4	58	4.34	3.11		0.191
		−4	44	30	5.16	3.43	54	0.211
		−12	50	30	4.47	3.16		0.195
		−42	8	38	5.95	3.70	34	0.225
Left Cingulate Gyrus	23	−2	−20	34	5.18	3.44	27	0.211
Right Cingulate Gyrus	32	12	22	36	4.65	3.24	22	0.200
		4	22	42	4.47	3.17		0.195
***PARIETAL LOBE***								
Left Superior Parietal Lobule	7	−34	−68	46	7.74	4.18	43	0.245
Right Supramarginal Gyrus	40	60	−48	22	7.23	4.06	24	0.241
***TEMPORAL LOBE***								
Left Middle Temporal Gyrus	39	−48	−60	24	5.72	3.63	36	0.222

**Table 3:** Regression analysis between brain activity during pain facial expressions observation and the “Personal Distress” subscale (uncorrected-*p* = 0.001; k = 20). Only negative relationships are represented because the positive ones did not reach statistical significance.

Region	BA	x	y	z	T	Z	Voxels/Cluster	r^2^
***PARIETAL LOBE***								
Left posterior cingulate gyrus	31	−14	−44	32	5.05	3.39	25	0.506

**Table 4:** Regression analysis between the “Empathic Concern” subtest score and functional connectivity of mirror neuron system regions (corrected *p* < 0.05 at cluster level; k = 40). Only positive relationships are represented because negative ones did not reach statistical significance.

Region	BA	x	y	z	T	Z	Voxels/Cluster
**Seed: Left IFG**							
Left Fusiform Gyrus	20	−51	−21	−30	4.01	3.32	49
Right Supplementary Motor Area	6	0	39	51	4.39	3.54	48
		3	51	45	4.03	3.33	
		3	57	39	3.43	2.95	
Right Middle Temporal Gyrus	21	69	−30	−12	4.05	3.34	57
		66	−33	−24	3.95	3.28	
		57	−36	−15	3.87	3.23	
**Seed: Left IPL**							
Left Superior Frontal Gyrus	10	−18	66	−6	7.07	4.76	161
		−18	69	15	5.44	4.08	
Right Superior Frontal Gyrus	10	18	63	−6	6.73	4.64	89
		15	69	3	4.72	3.72	
		24	66	−12	4.61	3.66	
Right Superior Temporal Gyrus	22	57	12	−3	5.18	3.96	119
Right Inferior Frontal Gyrus	45	42	42	−6	4.85	3.79	
		48	36	−6	4.73	3.73	
**Seed: Right IPL**							
Right Superior Frontal Gyrus	10	15	66	−6	5.85	4.27	65
		24	63	−9	4.80	3.77	
		15	66	3	4.51	3.61	
Left Superior Frontal Gyrus	10	−18	63	−6	5.46	4.10	122
		−33	60	−12	4.28	3.48	
		−15	66	15	4.48	3.59	43
		−9	66	21	4.46	3.58	
		−24	66	9	4.14	3.39	
Right Middle Frontal Gyrus	10	30	36	−3	3.89	3.25	88
Right Inferior Frontal Gyrus	45	48	39	−3	3.85	3.22	
		48	48	−6	3.68	3.11	

## Data Availability

The datasets that support the findings of this study are available from the corresponding author upon reasonable request.

## Supplementary Materials

The following supporting information can be downloaded at: https://www.mdpi.com/article/10.3390/bs13110947/s1, Table S1: ROIs analysis results; Table S2: Regression analysis between activity in the mirror neuron system activity region of interest (ROI) and the Interpersonal Reactivity Index (IRI) subscales.
