# Supplementary material for: Empathy Modulates the Activity of the Sensorimotor Mirror Neuron System during Pain Observation
Source: Behav Sci (Basel). 2023 Nov 17;13(11):947. doi: 10.3390/bs13110947 (PMC10669321; doi:10.3390/bs13110947)
Supplement: Supplementary file 1 [file behavsci-13-00947-s001.zip › behavsci-2651818-supplementary.pdf]

Supplementary Material

Table S1. ROIs analysis results.

Contrast: "Pain observation" vs. *Control (WHOLE GROUP)*			
**ROI**	**t-statistic**	**Uncorrected p**	**Corrected p**
Left Angular Gyrus	3.94	0.000529	0.004228
Left Inferior Frontal Gyrus	2.31	0.016865	0.127218
Left Inferior Parietal Lobule	3.35	0.001882	0.014956
Left Supramarginal Gyrus	3.63	0.001034	0.008241
Right Angular Gyrus	0.96	0.175592	0.786628
Right Inferior Frontal Gyrus	1.05	0.154033	0.737682
Right Inferior Parietal Lobule	1.21	0.121457	0.6451
Right Supramarginal Gyrus	0.64	0.264868	0.914706
Contrast: "Pain observation" vs. *Control (Men* vs. *Women)*			
**ROI**	**t-statistic**	**Uncorrected p**	**Corrected p**
Left Angular Gyrus	0.02	0.493231	0.995650
Left Inferior Frontal Gyrus	0.50	0.310028	0.948637
Left Inferior Parietal Lobule	1.05	0.154141	0.737949
Left Supramarginal Gyrus	0.38	0.353028	0.969304
Right Angular Gyrus	-0.01	0.505461	0.996422
Right Inferior Frontal Gyrus	0.55	0.296036	0.939688
Right Inferior Parietal Lobule	0.37	0.356951	0.970762
Right Supramarginal Gyrus	-0.14	0.553548	0.998422
Contrast: "Pain observation" vs. *Control (Regression with "Perspective Taking" subscale)*			
**ROI**	**t-statistic**	**Uncorrected p**	**Corrected p**
Left Angular Gyrus	-1.59	0.927096	1.000000
Left Inferior Frontal Gyrus	-1.39	0.900560	1.000000
Left Inferior Parietal Lobule	-0.56	0.704234	0.999941
Left Supramarginal Gyrus	-0.95	0.815720	0.999999
Right Angular Gyrus	-1.61	0.928701	1.000000
Right Inferior Frontal Gyrus	-1.05	0.838879	1.000000
Right Inferior Parietal Lobule	-0.65	0.735021	0.999976
Right Supramarginal Gyrus	-1.71	0.939449	1.000000
Contrast: "Pain observation" vs. *Control (Regression with "Fantasy" subscale)*			
**ROI**	**t-statistic**	**Uncorrected p**	**Corrected p**
Left Angular Gyrus	-0.04	0.516548	0.997016
Left Inferior Frontal Gyrus	-0.06	0.521532	0.997253
Left Inferior Parietal Lobule	0.91	0.193446	0.820911
Left Supramarginal Gyrus	0.10	0.460919	0.992868
Right Angular Gyrus	0.04	0.482572	0.994862
Right Inferior Frontal Gyrus	1.03	0.165178	0.764088
Right Inferior Parietal Lobule	1.24	0.123264	0.650901
Right Supramarginal Gyrus	0.84	0.210284	0.848725
Contrast: "Pain facial expressions" vs. *Control (Regression with "Empathic Concern" subscale)*			
**ROI**	**t-statistic**	**Uncorrected p**	**Corrected p**
Left Angular Gyrus	3.35	0.004279	0.033722
Left Inferior Frontal Gyrus	3.28	0.004754	0.037402
Left Inferior Parietal Lobule	1.78	0.053984	0.358514
Left Supramarginal Gyrus	2.59	0.014502	0.110294

Right Angular Gyrus	2.89	0.008972	0.069561
Right Inferior Frontal Gyrus	2.32	0.022914	0.169268
Right Inferior Parietal Lobule	1.92	0.043716	0.300647
Right Supramarginal Gyrus	3.40	0.003923	0.030955
Contrast: "Pain facial expressions" vs. *Control (Regression with "Personal Distress" subscale)*			
**ROI**	**t-statistic**	**Uncorrected p**	**Corrected p**
Left Angular Gyrus	-1.16	0.861205	1.000000
Left Inferior Frontal Gyrus	0.31	0.383497	0.979132
Left Inferior Parietal Lobule	-1.33	0.892365	1.000000
Left Supramarginal Gyrus	-1.23	0.875668	1.000000
Right Angular Gyrus	-1.08	0.845040	1.000000
Right Inferior Frontal Gyrus	-0.53	0.694544	0.999924
Right Inferior Parietal Lobule	-1.01	0.831579	0.999999
Right Supramarginal Gyrus	-0.94	0.813250	0.999999

Table S2. Regression analysis between activity in the mirror neuron system activity region of interest (ROI) and the Interpersonal Reactivity Index (IRI) subscales.

ROI	IRI's Subscale	Beta	p-value
Left IFG	PT	-0.445	0.096
	FS	-0.205	0.400
	EC	0.745	0.012
	PD	-0.083	0.700
Left IPL	PT	-0.208	0.379
	FS	-0.099	0.655
	EC	0.794	0.005
	PD	-0.159	0.423
Left AG	PT	-0.289	0.270
	FS	0.117	0.630
	EC	0.536	0.059
	PD	-0.363	0.107
Left SMG	PT	-0.216	0.439
	FS	0.066	0.800
	EC	0.507	0.092
	PD	-0.230	0.329
Right IFG	PT	-0.342	0.223
	FS	-0.098	0.705
	EC	0.616	0.044
	PD	-0.182	0.435
Right IPL	PT	-0.017	0.951
	FS	0.352	0.196
	EC	0.136	0.640
	PD	-0.223	0.350
Right AG	PT	-0.468	0.089
	FS	0.119	0.632
	EC	0.504	0.078
	PD	-0.360	0.117
Right SMG	PT	-0.464	0.079
	FS	0.258	0.285
	EC	0.496	0.072
	PD	-0.336	0.126

PT: "Perspective Taking"; **FS:** "Fantasy"; **EC:** "Empathic Concern"; and **PD:** "Personal Distress.
